# Enabling Smart Air Conditioning by Sensor Development: A Review

**DOI:** 10.3390/s16122028

**Published:** 2016-11-30

**Authors:** Chin-Chi Cheng, Dasheng Lee

**Affiliations:** Department of Energy and Refrigerating Air-Conditioning Engineering, National Taipei University of Technology, Taipei 10608, Taiwan; newmanch@ntut.edu.tw

**Keywords:** thermo-fluidic sensors, occupancy detector, smart air conditioning, energy saving, thermal comfort

## Abstract

The study investigates the development of sensors, in particular the use of thermo-fluidic sensors and occupancy detectors, to achieve smart operation of air conditioning systems. Smart operation refers to the operation of air conditioners by the reinforcement of interaction to achieve both thermal comfort and energy efficiency. Sensors related to thermal comfort include those of temperature, humidity, and pressure and wind velocity anemometers. Improvements in their performance in the past years have been studied by a literature survey. Traditional occupancy detection using passive infra-red (PIR) sensors and novel methodologies using smartphones and wearable sensors are both discussed. Referring to the case studies summarized in this study, air conditioning energy savings are evaluated quantitatively. Results show that energy savings of air conditioners before 2000 was 11%, and 30% after 2000 by the integration of thermo-fluidic sensors and occupancy detectors. By utilizing wearable sensing to detect the human motions, metabolic rates and related information, the energy savings can reach up to 46.3% and keep the minimum change of predicted mean vote (∆PMV→0), which means there is no compromise in thermal comfort. This enables smart air conditioning to compensate for the large variations from person to person in terms of physiological and psychological satisfaction, and find an optimal temperature for everyone in a given space. However, this tendency should be evidenced by more experimental results in the future.

## 1. Introduction

Air conditioning systems adopt sensors, in particular thermo-fluidic sensors, for feedback control in order to provide comfort environments. Recently, the progressive growth was observed in physical sensors [1], such like the sensors for temperature, relative humidity, pressure, wind velocity, and volume flow rate etc. Size minimization came with advancement in semiconductor manufacturing technology. The most important is the use of integrated circuits (IC) to increase accuracy while converting measured data into digital signal. The digital signal can be transmitted via wired or wireless communication. Data from various thermo-fluidic sensors can be integrated to control air conditioners in an efficient way. 

In this study, the development of thermos-fluidic sensors for air conditioning systems is reviewed. How to apply IC-type sensors, construct a wireless sensor network (WSN) by wireless communication and convert all data into a single index for environmental control are also discussed. Since the main purpose of air conditioning is to provide a comfort environment for human, personal factors should be involved. The occupancy detection technology includes passive infra-red (PIR) detection and the newest development of wearable sensing should be integrated with thermos-fluidic sensors to enhance interaction between occupants and equipment. Thus yields the development of future smart air conditioning. 

Feature of a smart air conditioner is to compensate large variations from person to person in terms of physiological and psychological satisfaction and find an optimal temperature for everyone in a given space. While ensuring thermal comfort, energy consumption should be saved for protecting our environment. The energy savings of applying smart air conditioning are analyzed quantitatively by real case studies published from 1986 to 2016. Among these cases, influences on thermal comfort and the possibility of causing discomfort are also considered. More details are illustrated in following sections.

## 2. Development of Sensor Technology

### 2.1. The Development of Thermo-Fluidic Sensors

First, collection of environmental factor data by thermal-fluidic sensors and its development are discussed. Thermo-fluidic sensors are used in air conditioning applications. In terms of measuring temperature, thermocouples utilize a bimetal contact to induce a heat-initiated electromotive force, which is used to measure temperature. Resistive Temperature Detectors (RTDs) test temperature changes by utilizing the property of resistance change according to the temperature in platinum wires. Thermistors are sintered metal oxides. Their resistance is sensitive to temperature and can provide signals. Thermopile-based digital temperature sensors are alloys of polycrystalline silicon and aluminum produced by semiconductor production processes, and measures temperature through a heat-induced electromotive force. Infrared thermopile sensors use infrared to detect temperatures of heat-emitting objects. For humidity, there are ceramic capacitors and resistive humidity sensors. Resistive humidity sensors use porous ceramics or macromolecules to absorb humidity in the air. The resistance will change accordingly and humidity is thus measured. Dielectric-variation capacitor hygrometers have a resistor that is exposed to the air outside. Humidity can be measured by the changes in resistance.

For pressure, piezoelectric sensors and silicon pressure sensors use ceramic piezoelectric materials or the piezoelectric effects of semiconductors to measure pressure. In addition, there are devices that emit electric signals through diaphragm + LVDT and diaphragm + strain gauge designs. Pitot tubes are used to measure pressure by finding the difference between total pressure and static pressure. Devices that use a hot wire work are based on the principle of cooling down when fluids pass through the resistance wires. Flow rate signals are obtained under an environment of fixed and controlled temperature, and the change in power can be calculated from the feedback. Ultrasonic anemometers use the phrase difference of ultrasonic transmissions caused by air flow velocity changes to obtain high-resolution flow rate measurements. Particle imaging velocimetry (PIV) is a special method. The method uses high-speed cameras to record dispersed particles under two laser exposures. The flow field and velocity distribution can be obtained from analysis. A Venturi tube is used to measure flow rates in a tube by the pressure principles of Bernoulli’s principle. Another method is to measure flow in the tube by means of observing rotational speed change in paddle wheels when a fluid passes through it. High-resolution data can be obtained by analyzing frequency shifts of ultrasonic reflections inside the tube via an ultrasonic Doppler flow meter. Resolutions up to 1 μL/min can be obtained [2,3,4,5,6,7,8,9]. 

The focus of structural improvements of thermo-fluidic sensors in recent years has not been on improving sensitivity, but on using semiconductor designs such as transistor gates, semiconductor junctions, micro-structure sensors, and self-assemble membranes to incorporate ICs in the sensors. The performance is expected to be comparable to that of existing analog sensors, but the costs will be greatly reduced. This advantage will allow thermo-fluidic sensors to be widely applied in everyday use. The examples of temperature sensor technologies and temperature sensor IC developments in this study illustrate the history of the development, as well as the technological changes triggered, scope of innovation, and potential benefits trigged by that development.

### 2.2. The Development of Temperature Sensors

Temperature is the most measured physical parameter of heat. The topic of this paper, smart air conditioning, is heavily related to heat sensors. In 1990, developments in heat sensors have proved satisfactory for use in air conditioners in terms of accuracy, output linearity, and operational dynamics [10,11,12,13]. Taking RTDs for example, the principle behind them is using the property that the resistance of pure metals will change according to temperature. RTDs are often made of platinum probes and specifically of Pt100, which are temperature detecting bodies with a resistance of 100 Ω and a typical temperature coefficient of 4 Ω/°C. If a standard current of 2 mA passes through such a resistor, a sensitivity of 8 mV/°C can be obtained. Through conversion with an A/D converter, the detected temperature range is from −200 to 800 °C with an accuracy of ±0.4 °C. Apart from Pt100, thermocouples are also frequently used sensors in temperature measurement. Over 60% of industrial air conditioners and freezing devices adopted thermocouples as temperature sensors [14]. The working principle is obtaining electrical potential differences caused by the thermal expansion of two different metals when they are in contact. Depending on the metals used, the measurable temperature range could be from 0~1000 °C with an accuracy of 0.5% ±0.7 °C. Even though the accuracy is not as good as RTDs, thermocouples are widely used because they are cheaper and smaller. Another temperature sensor common in industry is the thermistor, which is also cheap and small in size. With an accuracy of ±0.5 °C it is more accurate than thermocouples, however, its large non-linear effect needs to be calibrated before applying it in a specified measurable temperature range. A powerful candidate that is also cheap and small in size is the thermopile-based IC sensor. It produces thermopiles by using semiconductor technology, and then measures temperature variations through the voltage changes between bases and emitters of transistors. It can be integrated with amplifier wiring to allow direct output of temperature sensing signals with an accuracy of ±0.3 °C. However, due to IC manufacturing process limitations, it has a smaller range of −40~150 °C, which is still suitable for air conditioners [15].

IC sensors adopt the voltage change between the base and emitter of standard transistors to detect temperature variation. As transistors are standard semiconductor parts, they can be mass produced at a low cost. Technological progress allow signal amplifiers to be integrated with temperature sensing chips for various forms of output, such as: 

Linear output: The output voltage and temperature have a linear relationship.

Critical point output: It is mainly used to test overheating and permits setting a preset temperature protection setting. Signals are output when the temperature is higher or lower than the critical point.

Digital output: Signal levels can be output according to the stability of temperature signals. Its accuracy is comparable to that of lab-level thermocouple RTDs [15]. For this reason, IC temperature sensors have risen to the market pioneer status from being an auxiliary part in temperature sensing devices. Its technological edge comes from the support of semiconductor technology with lab-grade accuracy and very low prices for mass applicability.

Infrared thermometers are made from deposing gold on one end of the thermopile. The electromotive forces trigged by the infrared absorption can be used to detect temperature without contact. The infrared ear thermometer is an example of its application in everyday life. This has created a new market in biotechnology. It can achieve an accuracy of ±0.5 °C by converting signals through OP Amp.

The above is a summary of the literature from between 1980 and 1990, which relates to the development of temperature sensors, especially thermo-fluidic sensors. Four sample products of air conditioning temperature control are studied. As they are mature products, they will be referred to as traditional air conditioning systems. The adopted sensors and their achieved temperature control accuracy are studied. The results are presented in Table 1.

Table 1 shows that most commercialized air conditioning products utilize IC sensors. The advantage is IC integration, volume minimization, and signalizing physical measurements on site. The signals are transmitted to the air conditioning system for feedback control design through wire communication, such like universal asynchronous receiver/transmitter (UART) or inter-integrated circuit (I2C), or wireless communication such like Wi-Fi or Zigbee. This is the reason that IC sensors have become the mainstream temperature sensor used in traditional air control system design.

Based on temperature sensor development in Table 1, the temperature control accuracy of RTDs, thermocouples, thermistors, or IC sensors greatly exceed what is needed for temperature control of contemporary air conditioners. However, human comfort is not solely dependent on the accuracy of temperature. It also relates to environmental physical parameters, such as relative humidity (RH), air flow velocity, and radiative temperature, and human exercise physiology and clothing insulation [16,17,18]. A sensor suitable for smart air conditioners, would vary with human comfort, not temperature only. Thus came the next stage of sensor development, which was thermal comfort sensor development.

### 2.3. The Development of Thermal Comfort Sensors

Apart from enabling IC integration of thermo-fluidic sensors, another advantage of utilizing semiconductor production technology is that multiple sensing items can be integrated onto a single unit. Traditional thermo-fluidic sensors have independent sensor architecture for each corresponding physical parameter, and the integration into a single unit was nearly impossible. The complex properties of the system made it impossible to be applied on control systems for measuring the multiple physical quantities. However, micro electro-mechanical systems (MEMS) technology allows manufacturing multiple sensors onto a single chip, or implementing multi-chip module (MCM) by semiconductor packaging technology. This semiconductor packaging technology developed extensively in the 1990s [19], and this realized the measurement of multiple physical quantities with a single chip. A typical application would be the development of thermal comfort sensors.

The human body’s perception of environment comfort is not solely dependent on temperature, but a combination of temperature, humidity, surrounding air velocity, and radiative temperature. However, due to the impossibility of measuring multiple physical quantities with a single chip, the traditional air conditioners would rely solely on temperature feedback for control. By using MEMS, an integrated comfort sensor for measuring temperature, humidity, air pressure, and air velocity was developed. In 2000, Kang et al. exhibited a module that integrated thermal resistors, a hygroscopic layer, piezo-resistors, and flow sensors together [20]. The module fully illustrated an IC-type thermal comfort sensor. The sensor’s applications were also discussed.

By using the comfort index, air conditioner control can make adjustments according to real temperature changes and the indoor environment. Energy wastage caused by adjustments based on temperature alone could be avoided. Contemporary experiments indicated that 10% of the power usage of air conditioners could be saved [21]. Using the aforementioned integrated IC sensor for comfort feedback of air conditioners could be a great solution to the current energy problem.

However, mass-scale industrial applications of thermal comfort sensors that integrate thermos-fluidic sensors are still rare. The main problem is the high technology barrier for integrating sensors. Potential solutions may include applying MEMS for measuring multiple physical quantities, integrated chip packaging technology, multi-chip modules, and measuring multiple physical quantities by using heat. Most importantly, the technology of integrating sensors should not only measure the multiple physical quantities on a single device, but also should focus on how to implement data fusion, calculate a cumulative index from the measured data, and provide a feedback control index. This is critical development for smart sensor technology. Thus, after 2000, the development of sensor networks was presented. It integrated the thermo-fluidic sensors and wireless communication that could send the collected data, such as temperature, humidity, pressure, and wind velocity, to the data server. These collected information could be utilized for producing a control feedback index.

### 2.4. Wireless Sensor Network Development for Measuring Thermal Comfort

The aforementioned development in IC sensors provided an interface for digital signal output via UART and I2C. Its combination with wireless communications formed WSN, which comprises various distributed sensors. The fundamental concept is that each individual sensor will send the measured raw data back to the central system for computing. The system will make controlling decisions based on the computed and analyzed results from the received data. The collected data would be stored in the database for making decisions or triggering warnings.

WSN research related to sensor design and modern control theory. It also related to optimize sensor configuration based on the Neyman-Pearson lemma for minimizing possibilities of error in WSN measurements. Related theories on the topic were abundant and comprehensive [22]. However, WSN came with a complex system because of the dense configuration of signal wires. Thus, its application had been limited to factory production lines, central controlling systems, alarm systems, or large machines like aircraft control. Advancement in network technology could give WSN a new face. The wireless transmission replaced complex wiring, and this would improve the practicality of WSN. Researches related to the robustness of WSN, data throughput, and the effects of delayed effect and signal loss on control systems began to sprout [23,24,25].

Widely discussed wireless network architectures included building automatic control network (BACNet), Zigbee, and ultra-wide band (UWB). For example, Zigbee’s application in sensor networks [26] was: ZigBee stacks, which were set based on the IEEE 802.15.4 standard. Zigbee stacks defined the protocol’s multiple access control (MAC), physical (PHY) layers, NWK, APL, and security services.

The IEEE 802.15.4 is a standard for low power wireless personal area network (LP-WPAN). Its PHY layer’s wireless radio supports two different RF signals, located within the waveband of 2450 and 868/915 MHz. The 2450 MHz waveband could provide a data rate of 250 kbps and 16 different message channels. In the 868/915 MHz waveband, the 868 MHz supports one message channel with a data rate of 20 kbps, and the 915 MHz waveband supports 10 message channels with data rates of 40 kbps. The MAC layer is responsible for data communication between the nodes. It is responsible for setting up the synchronization with the network, supporting the correlation, decorrelation, and the safety of the MAC layer. It can provide a reliable link and prevent collision between two devices. Different layers of ZigBee stacks communicate with 802.15.4 MAC through SAP. SAP refers to the interface of the service provided by a specific layer and its upper layer. Most layers of ZigBee stacks have two interfaces, which are the digital and the management physical interfaces, respectively. The purpose of the digital physical interface is to provide routine digital services required by the upper layer. The purpose of the management physical interface is to provide access mechanisms for accessing internal layer parameters, configurations, and management data. ZigBee’s security mechanisms are provided by the security services providing layer, and the overall security of the system is composed by the security class of each network. 

However, there is a limitation in the bandwidth of wireless network communication. Large-scale data transmission would consume a lot of power, increase equipment costs and decrease overall system efficiency. For this reason, embedding a processor at every sensing point for organizing and sending the smaller volume of the data is the key to practice a reliable WSN. For example, the advanced surveillance camera systems will automatically start to record the images when the image changes. When there is no change in image, recording this data would only waste memory. By the same logic, a smart WSN node embedded with a processor can decide the format of the signal to send back to the server depending on the variation scope of the measured physical parameters. If such quantities are stable, the processor could notify the system with a simple string, instead of sending large volumes of raw data. This could greatly save network resources and realize smart WSN.

Researches in recent years have focused on reusing energy to provide the distributed sensor nodes with a power supply in the space. Potential sources of energy include light or electromagnetic waves, which can be converted to supply power for the sensor [26,27]. These kinds of developments enable WSN to be more flexibly distributed without limitation of battery. Removing the battery could solve the problems of changing and safely disposing of it, and make the application of WSN more environmentally friendly.

From the development of thermo-fluidic sensor and WSN applications, sensors have progressed from simply measuring temperature to measuring thermal comfort with more physical parameters, such as humidity and wind velocity. However, an interactive sensing technology is benefit for developments of smart air conditioning. The interactive sensing technology is occupancy detection, which will be discussed in the next chapter.

## 3. The Development of Occupancy Detection Technology

Occupancy detection is critical for generating interaction for mechanical control. Reports of air conditioners using occupancy detection for energy consumption control started in 1986 [28]. The sensors adopted at that moment were the PIR kind, which are still common today. Since 1980, there was a great progress on sensors and its application on occupancy detection. A review paper [29] in 2015 illustrated the development of PIR, ultrasound, carbon dioxide based detection, and radio frequency identification sensors. This study also presented the application of smartphones and wearable sensing on occupancy detection from published papers in 2015–2016. The description is as follows:

### 3.1. PIR Sensor

PIR sensors can detect the heat emitted from the human body, because the human body radiates thermal energy at the wavelength of 9~10 micrometers. The PIR sensor module consists of a pyroelectric infrared detector with a built-in amplifier and a digital output circuit. It has 64 detection zones, including 16 Fresnel-lens arrays, to collect infrared radiation from the four quadrant surfaces of the PIR detector. PIR sensors are highly sensitive to human movement. The advantages include low energy consumption, cheapness, and ease of deployment in buildings. In 1986, occupancy detection was used for energy control [28]. In 2009, Delaney et al. [30] adopted PIR-based occupancy detection systems to measure energy wastage in lighting in case of non-occupancy. In 2014, Marinakis et al. [31] demonstrated an integrated system which utilized remote control technology for real-time monitoring of energy end-users by occupancy detection. Optimization functions were achieved for energy conservation at a supermarket in Greece. In the same year, commercial slogans of Japanese air conditioners focused on “Smart Eye” or “Eco Move Eye” [32], which utilized PIR sensors to detect human body movement. Then the air conditioner could adjust its operation modes such as cooling, cold air, or wind, depending on human movement. Furthermore, if the air conditioner was set at low-power consumption mode, it will turn off if no detection of human movement occurs within 30 min.

Even though there were more than 30 years of research on PIR sensors as the standard sensor for occupancy detection, there still existed a problem needed to be solved. PIR sensors would be deceived from false negative signals when the occupant remained still during the observation period. For example, if a person was sitting in a chair reading a book up to the next observation time, the PIR sensor would not detect the body movement and the false signal would yield error control to turn off the light or air conditioning. In order to solve this problem, additional sensors were implemented in the PIR. Several studies [33,34,35] proposed integrating PIR and other sensors by WSN. Different types of occupancy detection technologies were developed for replacing the PIR sensor.

### 3.2. Ultrasound Sensor

Ultrasound sensors could measure the distance and detect human movement in order to establish occupancy detection [29]. The bat is a good example to explain the function of ultrasound sensors. It identifies occupancy movements and the location through the reflected ultrasonic wave at the same time. However, the problem was that ultrasound decayed quickly in the air. A single sensor may not complete occupancy detection in large areas. Even home environments, such as the living room or the bedroom, may be too big for complete occupancy detection via ultrasounds, unless sensors can fly like bats. As that is an unlikely application of sensors, ultrasound sensors do not seem like a good replacement for PIR sensors.

### 3.3. Carbon Dioxide (CO_2_) Based Occupancy Detection

People emit different amounts of CO_2_ depending on their status or stationarity. Leephakpreeda et al. reported that the emission variation of CO_2_ changes with the activities in a quantitative way [36]. A sitting person would generate carbon dioxide at around 0.27 L/min. If the person was lifting or packing, the CO_2_ generation rate would rise up to 0.53 L/min. Thus levels of carbon dioxide could be used to detect the level of physical activity for the person in the room to establish interactive air conditioning control.

However, ventilation rates vary when the doors or windows of the room open. In this case, estimating the occupant number would be difficult, due to the nonlinear relationship between the emitted and extracted CO_2_ content. Therefore, for applying on the occupancy detection, other sensors may be required for assistance. 

### 3.4. Radio Frequency Identification

Occupancy detection via electromagnetic waves, instead of sound waves, advanced a lot between 2000 and 2010. There were two types of occupancy detection using radio frequency. One was “with tag”, and the well-known example was radio frequency identification device (RFID). The other was “without tag”. It’s also known as device-free localization (DFL) systems.

For RFID systems, the entities or humans were tracked or detected by carrying a device that back-scatters a radio frequency signal using an electromagnetic resonance structure. In most cases of application, the tag was not actively powered, but enabled by the electromagnetic waves. Radio frequency waves can effectively be spread in space, yielding a low cost and robust system, and generating real-time responses for occupancy detection. Lin and Lee reported innovative RFID applications for saving energy [37]. Hoque et al. presented a system for monitoring the sleeping status by a RFID system with a tag and an accelerometer [38]. Compared with other sensors, RFID system seemed to be an effective system for occupancy detection, and achieving accurate human motion monitoring.

Labeling the human body with a tag is still a difficult task. DFL was proposed as an effective human presence-detecting technology based on radio signals. It did not require the person to carry a tag. The fundamental principle of DFL was that the radio signals would be reflected, diffracted, scattered or absorbed by objects in the propagating paths. The human body is a good absorber of radio signals as it is 70% water, which causes shadowing effects. Human presence would be indicated by the shadowing effect. Youssef and Mah introduced a developed DFL system using Wi-Fi network [39]. It can indicate a static person in an area of 750 m^2^ with an average error of 6.74 m. They also pointed out that DFL worked well at the 2.45 GHz frequency, as this corresponded to the resonant frequency of water molecules, which was most content of the human body.

Even though using radio frequency identification is occupancy detection technology equipped with great potential, people still have doubts about the security of electromagnetic waves for the human body, as mentioned above. Thus, the related technologies have only appeared in papers. Except for specific occasions, such as commercial application on identifying cars [40], the application of RFID systems in sensors are extremely rare in commercial and residential buildings.

### 3.5. Smartphone and Wearable Sensing

RFID systems send out electromagnetic waves to detect occupancy in a space, which raises health concerns. However, the existing electromagnetic waves, such as the communication spectrum used by cell phones, or Bluetooth communication between cell phones and periphery devices, would be more acceptable out of habit. In addition, the emission of electromagnetic waves by such devices is highly regulated by international standards and local governments, and people are comfortable with it.

For that reason, occupancy detection adopting cell phone communication, especially smart phones and wearable devices, is highly feasible. Related researches also included utilizing smartphones to remotely turn on air conditioning, and smartphones and global positioning system (GPS) to determine the location of the person outdoors. When the person was about to reach home, the air conditioning system could turn on beforehand. The GPS could also confirm the person’s absence from the space, and remind them whether they have forgotten to turn off the air conditioning. In addition, air conditioners could be simulated as social media communication objects, and inform its operative state through interaction [41]. Wearable devices, such as smart watches, could be adopted to analyze the movement of the person in the air-conditioned environment, and monitor their sleeping habits at night by the built-in accelerometer. The air conditioner could adjust its operations according to the status of the customers [42]. More recent developments included using Bluetooth communication for location-based service. An iBeacon in the space could provide appropriate service based on the person’s current location data [43]. Smartphones and wearable sensing were used with PIR sensors to enhance interaction. An appropriate air conditioning procedure would be recommended according to GPS and PIR sensor. For example, if the person came into the door from exercising or walking outside, the PIR sensor would direct the cold air away from the person to avoid getting cold, due to direct contact with the cold air. 

The development from temperature sensors to thermal comfort sensors, integrated with occupancy detection technology, has enabled air conditioners to detect occupancy in rooms and consequently achieved interaction [44]. The generation of the smart air conditioners combined the application of sensors with occupancy technology development. This would become the topic of this paper: how to enable smart air conditioners by sensor development.

### 3.6. Other Sensors

Indoor air quality (IAQ) also affects human comfort. The indoor air typically contains pollutants, such as second-hand smoke, radon, mold, carbon monoxide (CO), CO_2_ and volatile organic compounds (VOCs). CO_2_ is a kind of bio-effluent. Humans are the main indoor source of CO_2_. As discussed above, CO_2_ concentrations could be employed for occupancy detection. In addition to CO_2_, indoor air typically contained dozens of VOCs from building materials, furnishings, equipment, cleaning products, combustion activities, human metabolism, and perfumes. Several chemical sensors could detect VOCs. Recently, the electronic nose (E-nose) for detecting odors of flavor was proposed as a universal detector for IAQ [45]. The e-nose was a detection system including three major parts: a sample delivery system, a detection system, and an analysis system. It was sensitive to all volatile molecules. Commonly used detecting devices included metal-oxide-semiconductor (MOSFET) devices, conducting polymers, polymer composites, quartz crystal microbalance, and surface acoustic wave (SAW). Different devices would generate various signals based on the perceived molecules. By the analysis system, IAQ could be measured at a very high sensitivity to femto-molar concentrations.

Particles in outdoor air could penetrate indoors and become pollution sources. Some particles may be generated by equipment, such as copy machines and printers. Particles may contain toxic chemicals, and cause allergic reactions or infectious diseases. The particles smaller than 2.5 μm in diameter may likely accumulate inside people’s lungs. One may notice the serious problem of PM_2.5_ in mainland China. Therefore, particle counters were developed to measure the number of particle pollutants and warn people.

However, IAQ measurements are only able to warn people passively. They are unable to actively solve the problem via air conditioning control or ventilation systems [46]. Subsequently, this study considered IAQ-related sensors, such as chemical sensors, e-noses, and particle counters as other sensors, and not a kind of the thermo-fluidic sensors or occupancy detectors used by mainstream air conditioners.

Saving energy is an important task for a smart air conditioner. Digital power meter for measuring energy usage was also suggested to be integrated with air conditioning systems. Similarly, these meters were mainly used to measure voltage and current, and not directly related to air conditioning control. They were also listed under the “other sensors” category.

## 4. Integrated Development for Smart Air Conditioner

Following the previous discussion on thermo-fluidic sensors and occupancy detection technology development, the development history of air conditioners and the integration development of both technologies for enabling smart air conditioners will be discussed here. 

Continuing with the rapid growth in global economy in 1980s, home air conditioners became popular among the inhabitants of hot and humid Asian regions seeking better living quality. The mainstream product was the window-type air conditioner with a compressor built into the same unit. The machine blew air through cold coils, and controlled the indoor temperature and humidity. Even though the machine was capable of cooling the indoors to a comfortable level, the compressor was placed just outside the window and often caused noise pollution. 

Till 1990, split-type air conditioners became the mainstream product. These air conditioners separated the compressor from the indoor unit for reducing noise. As for the indoor unit, a low-noise cross flow fan was used to deliver cold air to control temperature and humidity. This independently-controlled indoor fan was equipped with more fan speeds, wind directionality, and interactive devices. Thus, it was capable of being integrated with thermo-fluidic sensors and occupancy detection technology for realizing the future smart air conditioners.

**Figure 1:**
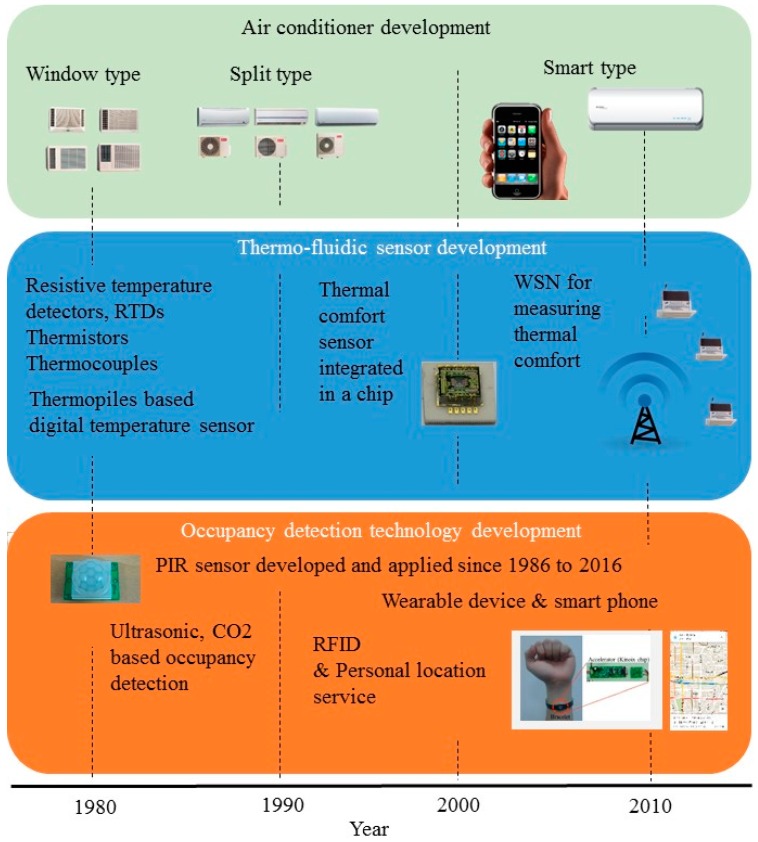
A brief history of the development of air conditioners, thermo-fluidic sensors and occupancy technology illustrating smart air conditioning and smart sensor development qualitatively.

Figure 1 presents the developing relationship among air conditioners, thermo-fluidic sensors and occupancy detection technology. While the air conditioner developed from window type, split type to smart air conditioners, the thermo-fluidic sensors also developed from temperature-control-based temperature sensors, thermal comfort sensors integrated in a single chip to dispersed WSN. One can notice that, because of sensor development, air conditioning control had evolved from simple temperature feedback to a thermal comfort index feedback, including temperature, humidity, wind velocity and radiant temperature. Regarding to the occupancy detection technology, PIR sensors have been developed and employed for air conditioning over 30 years. This technology has progressed from single sensors to sensor arrays, to the “Smart eye” [32] or “Thermo camera” [44], announced by the latest smart air conditioner for interactive control according to room temperature distribution, absolute location, and activity level of occupants.

Even when considering the same activity level and environment, people still have different perceptions of heat and the cold. Some don’t like the heat environment, others don’t like the cold one. The thermo-fluidic sensor for detection of environmental conditions and occupancy detectors for activity detection may still not provide a truly satisfying air conditioning environment. In order to satisfy individual requirements, smartphones and wearable devices could serve as the interactive interface. Wearable sensing technology, integrated with thermo-fluidic sensors and occupancy detection for enabling smart air conditioning, could serve as an interactive solution for providing everyone’s comfort.

## 5. Continuous Monitoring Technology with Multi-Parameters Sensors

Through the development of thermo-fluidic sensors, occupancy detection technology, smartphones and wearable devices for the smart air conditioning environment, information about the environment and occupancy are monitored and collected in the server or cloud system continuously for further analysis. The recent development history of continuous monitoring campaigns with multi-parameters sensors will be discussed here. 

The suitable air conditioning space is related to the state of the environment and the living style of occupants. Through continuously monitoring the state of environment, such as daylight [47], external temperature [48], added glazed façade [49], trigeneration energy system [50], system energy savings could be achieved. These systems need the assistance of simulation or computing software for precise calculation and estimation, such as daylight control system, IDA Indoor Climate and Energy software (IDA-ICE), building management systems (BMSs), etc. 

Long term monitoring of occupants’ movement may help understand one’s living style and create a personal air conditioning strategy. Development of low energy consuming wearable technology, such as Bluetooth Low Energy (BLE) [51], could fit the need for long term activity monitoring for healthcare and smart air conditioning. Through analyzing the peers’ personal attitudes on determining building thermal-energy, lighting performance, and openings’ schedules, one could find that occupants’ behavior would significantly affect building performance [52]. 

After long term monitoring of the environment and occupancy variation, huge amounts of data with certain noise or uncertain information would be collected in the server or cloud system. How to reduce the false information and disaggregate the integrated data, such as total power load or thermal comfort information, would decide the value of the collected data. Hence, the software or computing program, such as U_τ_ method [53] and Non-Intrusive Appliance Load Monitoring (NIALM) method [54], were developed. 

## 6. Case Study of Smart Air Conditioning

Previous sections have expounded the integrated development of thermo-fluidic sensors and occupancy detection technology, and how they provided an interactive control solution to achieve smart air conditioning. In this section, the quantitative energy savings and the achieved smart goals would be investigated by further literature survey. According to the database of the science direct on line (SDOL), the IEEE Xplore (IEL Online) and MDPI Open Access Journals platform, articles with the keywords that match “smart air conditioning” were searched. Among these articles, case studies were selected according to the following criteria:

- Applications related to “Thermo-fluidic sensor”.
- Applications related to “Occupancy detection”.
- The selected papers reported quantitative energy saving data.

**Figure 2:**
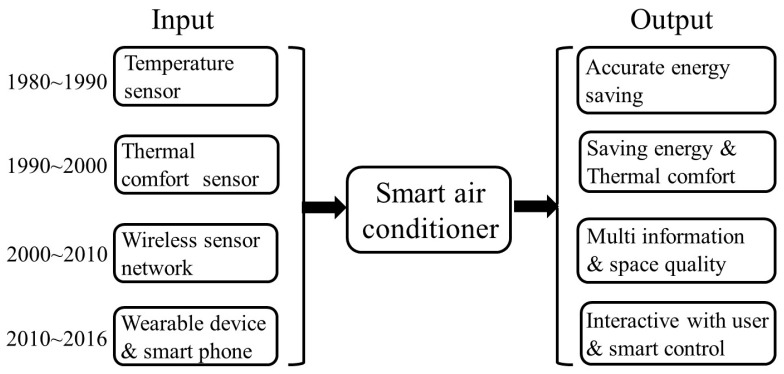
The qualitative analysis of the developed sensor technologies and achieved smart controls of the selected case studies in Table 2.

Table 2 summarizes the selected case studies. The developed sensor technologies and achieved smart controls of the selected case studies in Table 2 is summarized qualitatively in Figure 2. The developed sensor technologies for smart air conditioner includes temperature sensors (1980–1990), thermal comfort sensors (1990–2000), wireless sensor networks (2000–2010) and wearable device & smartphones (2010–2016), and these technologies caused the followed results for smart air conditioner, such as accurate energy saving, saving energy and thermal comfort, multi-information and space quality, and interactive with user and smart control, respectively. 

The 30 cases in Table 2 provided quantitative energy savings. All data would be put into the Equation (1):

(1) $$\mathrm{Energy}\;\mathrm{saving}\;\left(\%\right)=\left(1-\frac{\mathrm{Energy}\;\mathrm{usage}\;\mathrm{of}\;\mathrm{smart}\;\mathrm{air}\;\mathrm{conditioner}}{\mathrm{Energy}\;\mathrm{usage}\;\mathrm{of}\;\mathrm{original}\;\mathrm{one}}\right)\times 100$$

Quantitative energy savings could be used in discussing whether sensor improvement would significantly improve energy savings of air conditioner or not. Several experimental data, supplemented by several simulations or estimators from the published papers [60,61,64,70,76,77], were utilized to calculate the energy savings by Equation (1). This study simultaneously accounted the experimental and estimated data to evaluate energy saving performance based on sensor development. However, only the experimental data would be adopted for identifying the trend of energy savings.

Before identifying the trend quantitatively, the equipped sensors in each case would be discussed first. Comparing the thermo-fluidic sensor column in Table 2 with its development history in Table 1, one can notice that even though various researches had investigated the possibility of integrating thermal comfort sensors in a chip, there was no such real case. The widely discussed topic was the distribution-type WSN after 2011. Air flow or wind velocity sensors were the most often used sensors in combination with temperature sensors. Through enhancing ventilation or cooling around the human body, the set temperature points of air conditioners could be adjusted for saving energy. In the meanwhile, human comfort also could be ensured.

The enhancing ventilation, instead of intensive air conditioning, was an effective way to save energy. How sensors were applied to ensure thermal comfort could be discussed in details by thermal comfort theory, the PMV index [16,17,18,74], as presented in Equation (2):

(2) $$\begin{array}{l} \mathrm{PMV}=0.303e^{0.0036M}\\ \;+0.028\{\left(M-W\right)-3.05\times 10^{-3}\left[5733-6.99\left(M-W\right)\right]-P_{a}\\ \;-0.42\left[\left(M-W\right)-58.15\right]-1.7\times 10^{5}M\left(5867-P_{a}\right)-0.0014M\left(34-T_{a}\right)\\ \;-3.96\times 10^{-8}f_{cl}[{\left(T_{cl}+273\right)}^{4}-\left(T_{r}+273{)}^{4}\right]-f_{cl}h_{c}\left(T_{cl}-T_{a}\right)\} \end{array}$$

where $T_{cl}=35.7-0.028\left(M-W\right)-I_{cl}\left\{3.96\times 10^{-8}f_{cl}[{\left(T_{cl}+273\right)}^{4}-\left(T_{r}+273{)}^{4}\right]-f_{cl}h_{c}\left(T_{cl}-T_{a}\right)\right\}$.

The thermal comfort index, PMV, can be calculated by the above equation. This value depends on: (1) metabolic rate of human bodies, *M*, in unit of Wm^−2^; (2) the outward work rate, *W*, in unit of Wm^−2^; (3) the surface coefficient of clothes, *f_cl_*, in unit of m^2^·°C·W^−1^; (4) the insulation value of clothing, *I_cl_*, in unit of m^2^·°C·W^−1^; (5) the partial vapor pressure, *P_a_*, in unit of Pascal; (6) room temperature, *T_a_*, in unit of °C; (7) irradiation temperature, *T_r_*, in unit of °C; (8) clothing surface temperature, *T_cl_*, in unit of °C; (9) the convective heat loss coefficient, *h_c_*, in unit of Wm^−2^·°C^−1^; and (10) air flow velocity for determining *h_c_* according to the laminar or turbulent flow field.

The PMV value approaching zero means that the space is in the best comfort level for people to stay. Based on Equation (2), this condition may be met when the metabolic rate, *M*, is almost equal to the outward work rate, *W*. However, under normal air conditioned environments, the value *W* is not high. Hence, for the internal air to reach an equilibrium of heat dissipation, the difference between *W* and $f_{cl}h_{c}\left(T_{cl}-T_{a}\right)$ must approach zero. The *h_c_* value would be high when the indoor wind velocity is high. Thus, the indoor temperature *T_a_* does not need to be low for maintaining a fixed heat dissipation rate, while striking a comfortable equilibrium with the body’s metabolic rate *M*. This is the working principle of enhanced ventilation instead of air conditioning, which was discussed in an application case in 1992 [60]. This working principle was utilized to change users’ decisions about when and how to operate room air conditioners. Other case studies also widely adopted the combination of temperature sensor with air flow (wind velocity) sensor for reaching smart control [61,64,66,68,69,71,73,75,77,79]. Based on Table 2, all the cases, which achieved smart goals of building thermal comfort model and ensured occupancy comfort, employed air flow (wind velocity) sensors for enabling smart control. In addition, the effect of the humidity, *P_a_*, in the space as well as thermal radiation $f_{cl}[{\left(T_{cl}+273\right)}^{4}-{\left(T_{r}+273\right)}^{4}]$ needs to be detected and controlled through thermo-fluidic sensors, including humidity sensors [61,63,70,74,75,82,83] and thermal radiation sensors for improving overall comfort.

In summary, if the temperature, humidity, wind velocity, thermal radiation, human metabolic rate, outward work rate and clothing properties are detectable and controllable, it is possible to make the environment comfortable by adjusting air conditioning system. In Table 2, it can be seen that the developed sensors were widely utilized for feedback these parameters by means of WSN from 1982 to 2016.

As for retrieving information of human metabolic rate, outward work rate, and clothing properties, the method related to occupancy detection was disclosed in Table 2. Human motion could be guessed [72,76,80] by elementary personnel calendar or schedule. This motion could also be sensed by PIR or IR cameras [28,42,62,68,73,74]. Researches between 1986 and 2014 were not able to find a way to effectively detect human skin and core temperatures for calculating PMV in Equation (2). Furthermore, it was also difficult to detect closing properties without using a camera [74]. However, the use of cameras was in conflict with personal privacy, which would not be suitable for general air conditioning scenarios. Thus, an obvious bottleneck existed in developing occupancy detection technology in terms of retrieving the data to calculate PMV and control energy consumption. It was not solved until 2014 when a research utilized wearable sensing and smartphones as the interactive interface. It could detect human motion, human skin or core temperature, and complete physiological data of the human. Through interacting with the smartphone, the user could input parameters, such as personal feeling, clothing properties, and their willingness, to the air conditioning system. In comparison with the usage surveys [58,64,66] between 1980 and 1990, the development of sensors after 2010 integrated mobile communication mechanisms, such as GSM, Wi-Fi and Bluetooth. The status of the persons and their perception about the air conditioned environment could be known at any time. Development of occupancy technology realized not only human motion detection, but also intention sensing, which enabled air conditioners to be smarter and smarter.

As for other sensors, one could note the development of digital power meters [82] and electricity frequency detectors [81] in Table 2. They were responsible for detecting the physical quantities related to currents and power consumption. This development could promote the integration of air conditioners and smart grids together in the future, and alleviate the problems of electricity shortage during peak loads.

Finally a development summary of the achieved smart air conditioning goals by using sensors development from 1982 to 2016, as illustrated in the last column of Table 2, is presented. From 1982 to 1986, the main topics consisted of using computer or other hardware digital equipment to receive and integrate signals for different kinds of sensors. From 1996 to 1999, the main progress was using these detected signals to achieve optimization of air conditioners to save energy. In 2001, the concept of human comfort, whose goal was to maintain human comfort while saving energy, was introduced. In 2005, personalized air conditioning system appeared. Sensors were not only used for controlling air conditioning equipment, but also assessing human thermal comfort. Cumulative discussions on the CFD model and bio-heat were published in 2016. In 2014, the smart phone and wearable device were utilized for sensing human comfort. In summary, the study cases from 1982 to 2016 indicated the developing trend of smart air conditioners and related sensors. Smart air conditioners now could interactive with users to ensure human comfort while saving energy.

## 7. Discussion

**Figure 3:**
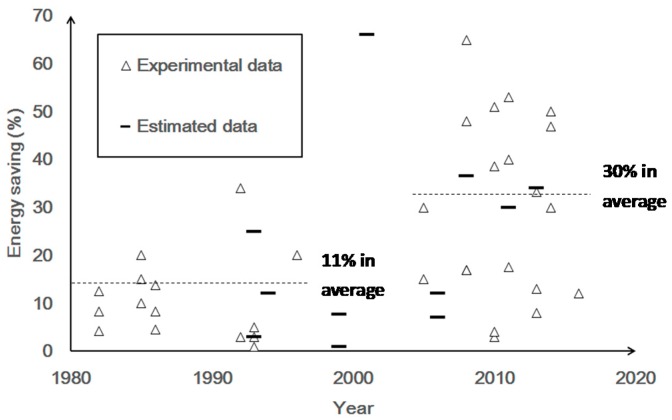
Energy savings (%) achieved by smart air conditioning researches, enabled by sensor and occupancy detection technology from 1982 to 2016.

The calculation of energy savings in Equation (1), as well as personal comfort of PMV index in Equation (2), are further discussed quantitatively in this section. The energy saving data of all cases presented in Table 2 could be separated into two types: estimated or experimental data. The estimated data came from estimation of simulation of air conditioner for obtaining energy savings. The experimental data came from actual smart air conditioner enabled by a sensor. The energy savings in %, based on Equation (1), with respect to required cases at each year were plotted, as shown in Figure 3. 

In Figure 3, the average energy savings of selected cases study before 2000 was 11%, with the standard deviation of 8.92%, and that after 2000 was 30%, with standard deviation of 18.65%. This variation indicated that, before 2000, the improvement of sensor performance caused lower energy savings. However, after the initiation of thermal comfort technologies, especially the mobile phone, smart phone and wearable devices, the energy savings increased significantly. In 2001, a research related to air conditioning control [65] discussed the possibility of increasing air speed instead of lowering temperature to ensure comfort. The estimated energy savings of 66% was reported and shown as a short rectangular line in 2001 in Figure 2. In 2008, a research related to human comfort improvement [69] empirically proved its energy savings up to 65% by increasing air movement and using RTDs with accuracy of 0.1 °C in temperature sensing and wind velocity sensors with resolution of <0.2 m/s. The air speed of the air conditioner was increased to offset the impact of increased room air temperature for occupants’ comfort. The offset of increasing temperature settings up to 4 °C enabled the air conditioner to save 65% of energy. Most of the important, by combining with the thermal comfort technologies, the proper thermal comfort (−0.5~0.5 of predicative mean vote, PMV) and energy savings could be achieved simultaneously. The results of combing the energy savings and thermal comfort control after 2005 would be analyzed further in the followings. 

**Figure 4:**
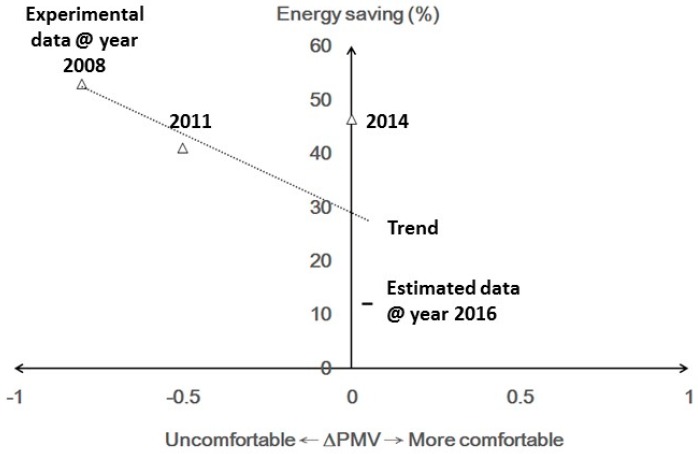
The aforementioned experimental data and the estimated data of energy savings of air conditioner through the development of fluidic sensors with occupancy detection technology from 2008 to 2016.

Despite the offset effect, a higher temperature setting of air conditioner still caused discomfort. How to save energy while ensuring thermal comfort is a question, and the developments of occupancy detection technology may have the answer. WSN and wearable sensing development had combined with PIR detectors since 2008. By using wireless sensors, mobile phones, or wearable devices, the human motion, as well as human intention, could be detected for controlling the smart air conditioner. This would be able to control the PMV index, as illustrated by Equation (2), in a certain range while approaching zero. According to reports of energy savings and improved comfort by increasing air movement in 2008 [69], as well as reported air conditioning system in Taiwanese convenience stores in 2011 [74], a −0.5~−0.8 decrease in the PMV value could save 41%~53% of consumed energy. In 2014, a bracelet equipped with accelerometer was used to detect the human’s sleeping state [42]. Once the human was asleep, the air conditioner could immediately be switched to fan mode for saving 46.3% of energy without human notice. This mean that energy could be saved while ∆PMV approaching 0. Comparing with the previous applications of thermo-fluidic sensors for controlling smart air conditioning, the combination of fluidic sensors with occupancy detection would provide a total solution for saving energy and ensuring human comfort. The aforementioned experimental data and the estimated data from reference [83] was presented in Figure 4. 

In Figure 4, the smart air conditioner progressed from −1 of ∆PMV, an uncomfortable environment [69], to a comfortable one of 0 of ∆PMV [42] and achieved energy savings of 46.3% by using wearable sensing to sense the human body. In 2016, the wearable sensing was integrated with multi-evaporator systems, providing the distribution of cooling evaporators around occupancies, as well as rapid adjustments of wind speed and air conditioning temperature according to human metabolic rate [83]. The energy savings of 10% could be achieved while keeping the thermal comfort improvement up to 0.1. Based on the collected data, the regressed trend may indicate that sensor development would enable air conditioners to maintain human comfort while saving energy. This would be evidenced by more experimental results in the future. 

## 8. Conclusions

This paper surveyed the development of thermo-fluidic sensors, occupancy detection technology, and the integration for enabling smart air conditioning. The effects of energy savings and ensuring thermal comfort are also discussed by application cases. According to the above discussions, six main points of conclusions could be made as follows:

- Energy savings achieved by air conditioning combined with thermo-fluidic sensors and occupancy detection technology has increased annually from 1982 to 2016. Before 2000, the average energy savings was only around 11%. After 2000, the average energy savings increased up to 30%. These results indicated that the sensor development successfully enabled smart air conditioning to save energy effectively.
- Sensor development also benefitted thermal comfort apart from energy saving. By using wearable sensing devices to detect the human body, an uncomfortable environment of ∆PMV approaching −1, caused by energy saving, could be improved to a comfortable one, ∆PMV approaching 0, with energy savings of 46.3%.
- Thermo-fluidic sensors could be evolved not only for measuring temperature, but also sensing the thermal comfort. According to the PMV formula in the study, *T_a_* could be increased by increasing *h_c_* for keeping the term $f_{cl}h_{c}\left(T_{cl}-T_{a}\right)$ constant. Therefore, energy could be reserved by increasing both air speed and temperature. This is an elementary method in air conditioning control.
- From the sensor developing history, it was recommended that thermo-fluidic sensors be integrated onto a single chip. However, the better way to use thermo-fluidic sensors from all case studies was for WSN, which could be dispersed in space to collect parameters related to human comfort, such as temperature, humidity, wind velocity, and thermal radiation. The parameters could enable the air conditioner to operate in a more energy-saving pattern.
- The application of occupancy detection technology on air conditioners was based on PIR sensors since 1980. Later on the array type sensors were presented, and IR detectors were replaced by thermo-cameras. These were more efficient in detecting the human location, and could direct the conditioned air towards the person. For a general scenario, when a person just entered the room or was sweaty because of doing sports, a discomfort situation could be avoided by redirecting the air away from the person via PIR sensor feedback.

The most interesting development of occupancy detection technology in recent years was wearable sensing. Wearable sensing devices could detect human motion up close, even human skin or core temperature, and collect human physiological data. Through interacting with smartphones, the user could enter information, such as personal feeling, clothing properties, and their mood, into the air conditioning system. Hence, the status of the person could be known at any time, and the air conditioning system could be adjusted to cater to fit the human needs better. In summary, through the recent published results, the occupancy detection technology could realize not only human motion detection, but also intention sensing for air conditioners to be smarter and smarter. This tendency would be evidenced by more experimental results in the future. 

## Figures and Tables

**Table 1:** Sensors employed by traditional air conditioning system and achieved air conditioning temperature control.

Product	Sensor Type	Range	Accuracy
H-brand temperature control (USA)	IC sensor	5~30 °C	0.5 °C
NI-209 temperature control (Taiwan)	IC sensor	−40~50 °C	1 °C
S-brand temperature control (Japan)	1000 Ω Platinum	−20~70 °C	0.5 °C
S-brand temperature control (Germany)	IC sensor	0~50 °C	1 °C

**Table 2:** Sensors employed by novel air conditioning system and achieved smart control.

Year	Air Conditioning Case	Equipped Sensors			Achieved Smart Control
		Thermo-Fluidic	Occupancy Detection	Others	
1982–1983	EMS for Heating/ventilating/air conditioning equipment: Case study of USA [55,56]	Thermocouples + Multiplexor + Minicomputer system	X	X	Using a computer for supervisory control allows the equipment to be operated in a more efficient manner through temperature sensor feedback controls
1985	Energy management for air conditioning system in Kuwait [57]	RTDs with accuracy to 0.1 °C temperature sensing	X	Water flow rate sensor	Energy management and economic analysis based on occupancy periods and the present values of life-cycle costs
1986	Computerized energy management system installed in the small to large industries and campus type facilities [28]	RTDs + Micro- and minicomputers with 4–10 floating per unit	PIR sensor + hardware digital equipment	X	Hardware digital equipment with occupancy detection function for start/stop of equipment and stand-alone demand controller
1986	Thermostat management for reducing household energy [58]	Thermostat based on thermistor	Home ownership investigation	X	Self-reported winter and summer thermostat settings and control strategies according to sensor data and occupancy status
1992	Users’ decisions about when and how to operate room air conditioners [59]	Thermostat based on thermistor + Wind velocity indicator	X	X	By user education, resident can operate air conditioner by non-thermostatic mode.
1993	Energy management for multi-zone air conditioning systems in Canada [60]	Thermistors with accuracy to 0.5 °C temperature sensing	X	Disturbance input	Multi-zone control based temperature sensor and disturbance signal
1994	A two zone variable air volume system [61]	Supply, return, entering and leaving air condition sensors include air density, velocity, temperature and humidity	Input data related to occupied period	X	A reduced model for variable air volume system to account mass, momentum and energy balance for saving energy
1994	Comfort control for short term occupancy at hotel [62]	Thermostat based on thermistor	PIR sensor integrated in a prototype ‘comfortstat’	X	Interactive set-point adjustment with immediate response to thermal requests
1996	Optimization of thermal processes in a variable air volume system [63]	Thermistors with accuracy to 0.5 °C temperature sensing + Humidity sensor	Thermal load prediction	X	Optimized thermal processes to achieve thermal comfort by both zone temperature and humidity ratio
1999	On-line control strategies for air conditioning system [64]	RTDs with accuracy to 0.1 °C temperature sensing + Air flow rate sensor + Pressure sensor	Investigating number of occupants	CO_2_ sensor	Optimizing pressure set-point of variable air volume system to achieve thermal comfort and improve air quality
2001	Air conditioning control to ensure comfort [65]	RTDs with accuracy to 0.01 °C temperature sensing	CO_2_ detection for improved start-stop time control	Integrated IAQ sensor	Air bypass, CO_2_ control, setback and improved start-stop time
2005	Personalized ventilation for air conditioning in a hot and humid climate [66]	Thermistors with accuracy to 0.5 °C temperature sensing + Air flow rate sensor with resolution to 0.01 L/s	Investigating number of occupants and detailed data includes sex, age, height and weight	X	Personalized ventilation to improve the immediate breathing zones of occupants in the built environment
2006	Optimal set point strategy to achieve energy efficient operation of air conditioning system [67]	Thermistors with accuracy to 0.5 °C temperature sensing	Occupied time adaptive controller based year-month-day function	X	Occupied time adaptive control and energy efficiency through optimal set point
2008	Energy saving and improved comfort by increased air movement [68]	RTDs with accuracy to 0.1 °C temperature sensing + wind velocity sensor with resolution <0.2 m/s	X	X	Elevating air speed which can offset the impact of increased room air temperature on occupants’ comfort
2008	Enthalpy estimation for thermal comfort and energy saving in air conditioning system [69]	Thermistors with accuracy to 0.5 °C temperature sensing + Humidity sensor for estimation Enthalpy	Optimum operative temperature for people during light, primarily sedentary activity	X	The least enthalpy estimator combines the concept of human thermal comfort with the theory enthalpy
2010	Task ambient conditioning system [70]	Thermo-camera with accuracy to 1 °C temperature sensing + wind velocity sensor with resolution to 0.5 m/s	Infra-Red images	X	A special air conditioning system heats only the feet and hands, and cools only the hands and face, to provide thermal comfort
2010	Air conditioning system of an AHU dedicated to the personalized ventilation system and an overhead fan-coil dedicated to control the room air temperature [71]	Thermistors with accuracy to 0.5 °C temperature sensing + Air flow rate sensor with resolution to 0.1 m^3^/s	X	X	Microclimate control by an individually controlled air distribution system aimed at improving the quality of inhaled air and thermal comfort off each occupant
2010	Campus air conditioning system managed by control center on internet [72]	RTDs with accuracy to 0.01 °C temperature sensing	Scheduled time-of-day	X	Scheduled control for energy saving
2010	Ceiling mounted personalized ventilation system [73]	Thermistors with accuracy to 0.5 °C temperature sensing + Air flow rate sensor with resolution to 1 L/s	PIR sensor	X	Using desk fans for providing convection cooling to each occupant in rooms
2011	Air conditioning system in conveniences stores in Taiwan [74]	IC type temperature sensor + embedded system for constructing a WSN	Digital camera	Digital power meter	WSN provides feedback of distributed thermal comfort index and controls environment
2011	Chilled ceiling and displacement ventilation aided with personalized evaporative cooler [75]	Thermistors with accuracy to 0.5 °C temperature sensing + Air flow rate sensor with resolution to 0.1 L/s + Humidity sensor	Personal location service	X	Personalized air conditioning directly towards the occupant trunk and face
2011	Air conditioning system strategies for energy conservation in commercial buildings in Saudi Abraia [76]	Thermostat based on thermistor	Specified schedules	X	Air conditioning model verification, investigation of energy savings and thermal comfort
2013	Personalized air condition and desk fan control for the convection flow around occupants [77]	Temperature sensor with accuracy to 0.01 °C + Thermal radiation sensor + wind velocity sensor with resolution to 0.1 m/s	Skin and core temperature; Sensible and latent heat; Clothing properties; Human metabolic	X	Building three models: CFD model; Thermal comfort model; Multi-segmental bio-heat model
2013	A versatile energy management system for large integrated cooling systems [78]	Thermistors with accuracy to 0.5 °C temperature sensing + Ambient property sensor	X	Level sensor	Versatile energy management platform for energy saving control of four large cooling systems
2013	A low-mixing ceiling mounted personalized air conditioning system [79]	Thermistors with accuracy to 0.5 °C temperature sensing + Air flow rate sensor with resolution to 0.1 L/s	Location based service	CO_2_ sensor	CFD, bio-heat, and comfort model coupling
2014	Variable air volume air conditioning system for buildings with large number of zones [80]	Thermostat based on thermistor	Calendar for occupancy prediction	X	Model predictive control
2014	Smart sensors enabled smart air condition control [42]	IC type temperature sensor	PIR detector, mobile phone and wearable device	X	Wearable sensing for smart control
2015	Supervisory control methodology for air condition system of commercial buildings [81]	Thermistors with accuracy to 0.5 °C temperature sensing	X	Electricity frequency detector	Air conditioning control to electricity grid integration
2016	Indoor air quality and energy management through real-time sensing in commercial buildings [82]	Thermistors with accuracy to 0.5 °C temperature sensing + Humidity sensor	Occupancy/movement detecting system through Wifi, GSM or Bluetooth signals, or through volume recognition with depth sensors (Ultrasound sensor)	Digital power meter	CFD, bio-heat, and comfort model coupling
2016	Multi-evaporator system integrated with networked control systems in large spatially distributed plants [83]	IC type temperature sensor + Embedded system for constructing a WSAN (Wireless sensor and actuators network)	Evaporator assembled near crowds in many places	X	Completing a detailed analysis of the end-to-end real-time flows over WSAN and a real-time kernel with an earliest deadline first (EDF) scheduler

## Nomenclature

BACnet	Building automation control network
CO	Carbon monoxide
CO_2_	Carbon dioxide
DFL	Device-free location
EDF	Earliest deadline first
E-nose	Electronic nose
GPS	Global positioning system
IAQ	Indoor air quality
LP-WPAN	Low power wireless personal area network
MAC	Multiple access control layer
MCM	Multi-chip module
MEMS	Micro electro-mechanical systems
MOSFET	Metal-oxide-semiconductor field electronic transistor
PHY	Physical layer
PIR	Passive Infra-Red
PIV	Particle imaging velocimetry
PMV	Predicted mean vote
RFID	Radio frequency identification device
RH	Relative humidity
RTDs	Resistive temperature detectors
SAW	Surface acoustic wave
SDOL	Science direct on line
UART	Universal asynchronous receiver/transmitter
UWB	Ultra-wide band
VOCs	Volatile organic compounds
WSAN	Wireless sensor and actuators network
